# Acquired hemophilia A that required surgical hemostasis of hematomas occupying oral cavity: a case report

**DOI:** 10.1186/s13256-021-02669-w

**Published:** 2021-02-15

**Authors:** Keigo Maeda, Shinsuke Yamamoto, Naoki Taniike, Toshihiko Takenobu

**Affiliations:** 1 Department of Oral and Maxillofacial Surgery, Kobe City Medical Center General Hospital, 2-1-1, Minatojima Minamimachi, Chuo-ku, Kobe-City, Hyogo 650-0047 Japan; 2 Department of Fixed Prosthodontics and Occlusion, Osaka Dental University, 1-5-17, Otemae, Chuo-Ku, Osaka-City, Osaka 540-0008 Japan

**Keywords:** Acquired hemophilia A, Autoantibodies against factor VIII, Intraoral hematoma, Recombinant activated factor VII, Surgical hemostasis

## Abstract

**Background:**

Acquired hemophilia A is a rare coagulopathy caused by inhibitors of blood coagulation factor VIII. Patients with acquired hemophilia A have a higher mortality risk (5–10%) than those with congenital hemophilia. Moreover, there is no established evidence of management recommended for patients with acquired hemophilia A. Previous studies have reported the presence of hematomas in the oral cavities of patients with acquired hemophilia A, which were treated conservatively. Here, we describe the case of a patient with acquired hemophilia A, where emergency surgical hemostasis was required for large intraoral hematomas.

**Case presentation:**

A 65-year-old Japanese man was referred to our hospital with a chief complaint of bleeding from large intraoral hematomas. On examination, he could not close his mouth because of the hematomas, which were bleeding spontaneously. Computed tomography angiography revealed no evidence of arteriovenous malformation, and blood test results showed that the activated partial thromboplastin time was elevated beyond the normal limit. To avoid a life-threatening hemorrhage from hematomas, emergency surgical hemostasis was performed with nasotracheal intubation using fiberoptic bronchoscopy. Hemostasis was successfully performed, as the hematomas were carefully removed. Moreover, the clinical course was successfully completed using intravenously administered activated prothrombin complex concentrate for hemostasis after operation.

**Conclusions:**

Acquired hemophilia A can cause a life-threatening hemorrhage without predictive factors. Intraoral hematoma may cause airway obstruction. There is no consensus regarding the management of hemorrhage in patients with acquired hemophilia A. As shown here, exophytic hematomas in the oral cavity can be safely removed and nasotracheal intubation with fiberoptic bronchoscopy may be useful in patients with coagulopathy disease.

## Background

Acquired hemophilia A (AHA) is a rare, acquired coagulopathy, resulting from autoantibodies against autologous factor VIII (FVIII). The incidence of AHA is estimated to be approximately 1.5 cases per one million individuals per year [1]. Some clinical presentations of AHA may differ from those of congenital hemophilia, as AHA is typically found in patients aged > 65 years, whereas there is no difference in the sex ratio [2]. Subcutaneous purpura and muscle hematoma are typical clinical manifestations in patients with AHA; however, hemarthrosis, which is common in patients with congenital hemophilia, is rare in those with AHA [3]. Some AHA cases are detected incidentally, as they can present with mild or no significant bleeding [4]. However, AHA is a more serious disease than congenital hemophilia. Moreover, emergency hemostasis or transfusions are required in 70–90% of the patients, and the mortality rate is 5–10% [5]. Hence, careful management is required. There have been some reports of hematomas that developed in the oral cavities of patients with AHA, which were treated conservatively [6–9]. However, there have been no reported cases of hematomas managed with surgical hemostasis. Herein, we report the case of a patient with AHA that required emergency surgical hemostasis for large intraoral hematomas.

## Case presentation

A 65-year-old Japanese man was hospitalized at another general hospital to undergo a bypass surgery for left middle cerebral artery stenosis. A few weeks before hospitalization, he had an ulcer on the oral mucosa, which sometimes oozed with blood; however, hemostasis was achieved spontaneously. After hospitalization, large hematomas with continuous hemorrhage developed in his oral cavity; therefore, he was transferred to our hospital for hematomas management. According to the physicians at the previous hospital, the patient had a history of hypertension and diabetes mellitus with chronic kidney disease (not requiring dialysis), and his activated partial thromboplastin time (aPTT) had been increasing over the past 3 months.

**Fig. 1:**
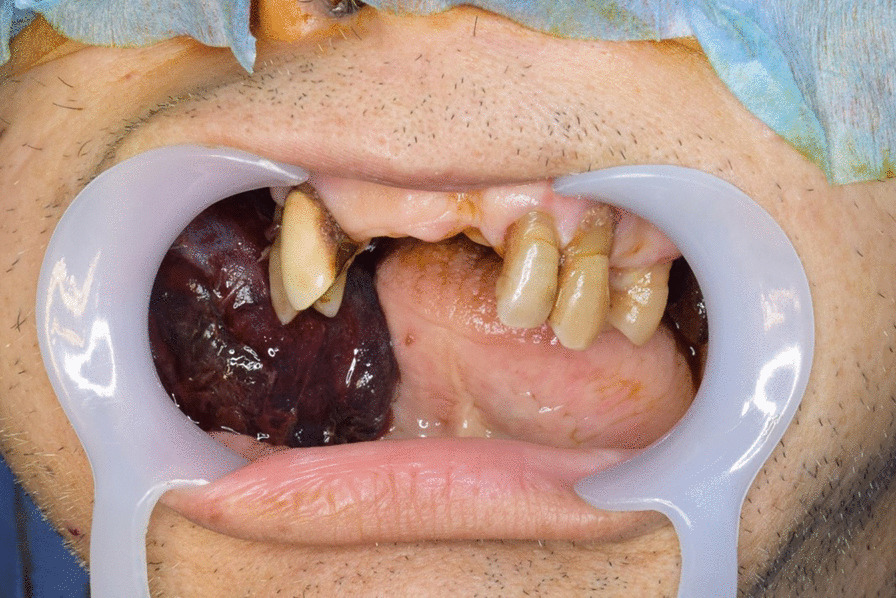
Preoperative appearance of large hematomas in the oral cavity. The hematomas were approximately 60 (right buccal mucosa) and 30 mm in diameter (left buccal mucosa)

**Fig. 2:**
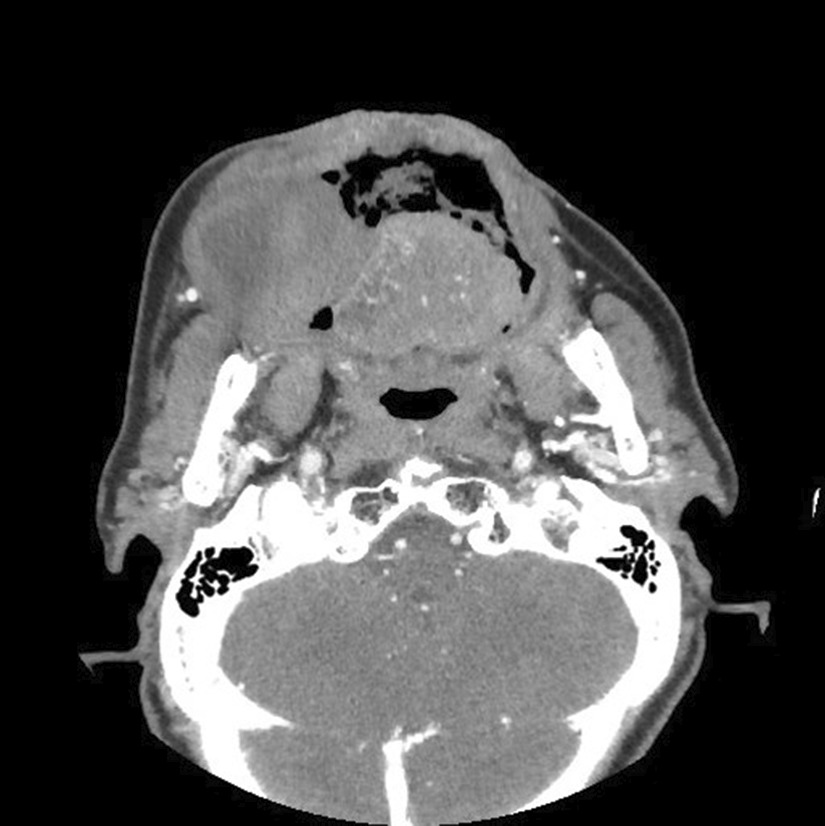
Computed tomography angiogram of the hematomas showing a large hematomas in the right buccal mucosa, with no evidence of arteriovenous malformation

On examination, his consciousness was clear, and his vital signs were within the normal limits. No purpura was observed on the entire skin surface. His face had no swellings, and no other significant findings were observed; however, he could not close his mouth because of the presence of two large intraoral hematomas that occupied the oral cavity. The hematomas were approximately 60 (right buccal mucosa) and 30 mm in diameter (left buccal mucosa) (Fig. 1). Laboratory tests revealed that his anemia had become more advanced since his admission to the previous hospital. He had a significantly prolonged aPTT of 84.8 seconds, a red blood cell count of 230 × 10^4^/µl, hemoglobin level of 7.4 g/dl, and hematocrit level of 21.7%. A computed tomography (CT) angiogram revealed no blood vessels flowing in or out of the hematomas, and there were no findings of arteriovenous malformations (Fig. 2). No obstructions or deviations were observed in the upper airway. After physical examination, coagulopathy was suspected, and an emergency surgical hemostasis was planned because of the risk of hematomas rupture and severe hemorrhage.

**Fig. 3:**
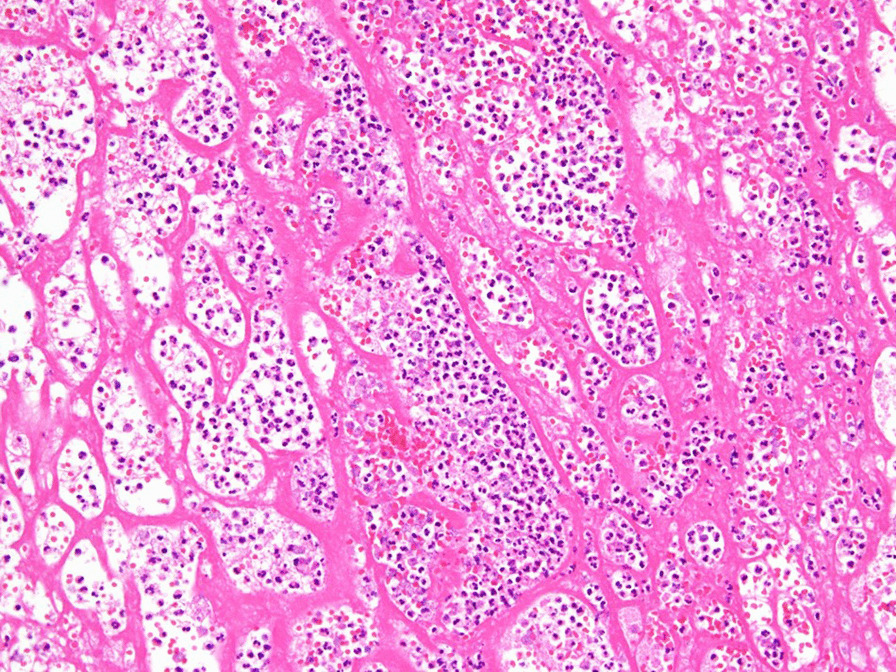
Histopathologic findings. Histopathologic findings of the lesion demonstrate that the hematomas contains fibrin, inflammatory cells, and bacterial congeries

We resected the intraoral hematomas and achieved surgical hemostasis under general anesthesia and nasal intubation with fiberoptic bronchoscopy. When the hematomas on both sides of the buccal mucosa were removed by careful blunt dissection, oozing of blood was observed and hemostasis was completed by cauterization. The wound in the right buccal mucosa was replaced by an artificial dermis, and that on the left was closed by reefing. The excised hematomas were sent for histological examination. The pathological findings showed no evidence of tumor in the hematomas (Fig. 3).

**Fig. 4:**
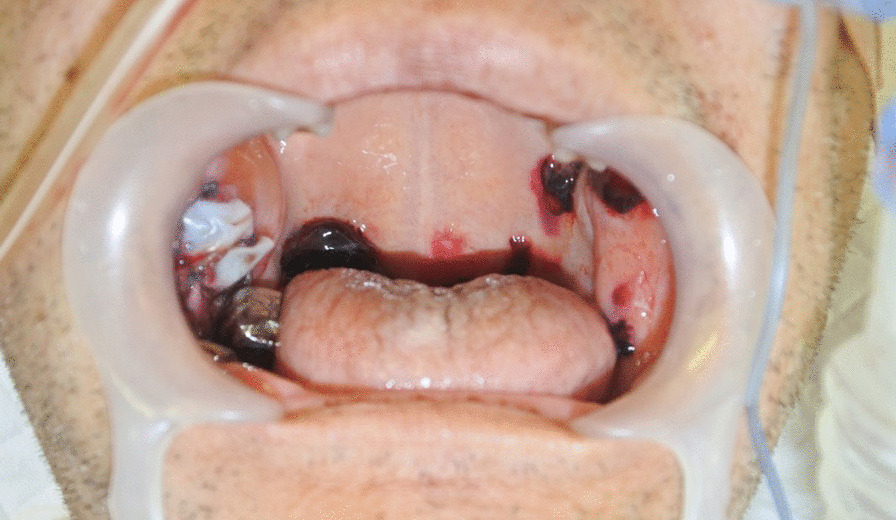
Postoperative appearance of a few hematomas in the velum. A few new hematomas of 10 mm diameter or less arose on both sides of the velum

**Fig. 5:**
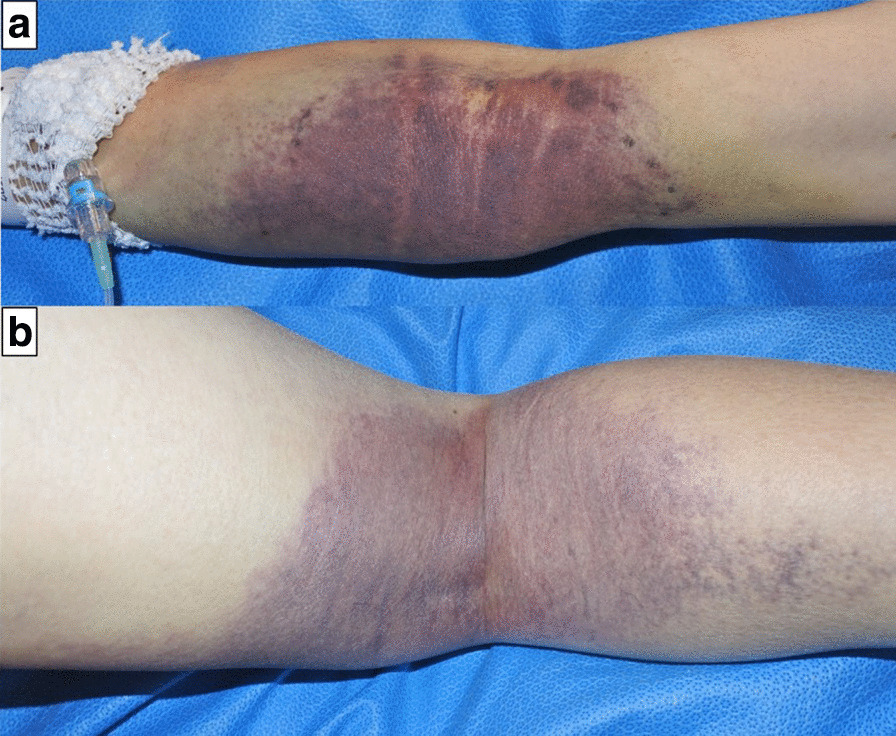
Purpura in the right upper and lower extremities. **a** Upper extremity; **b** lower extremity

**Fig. 6:**
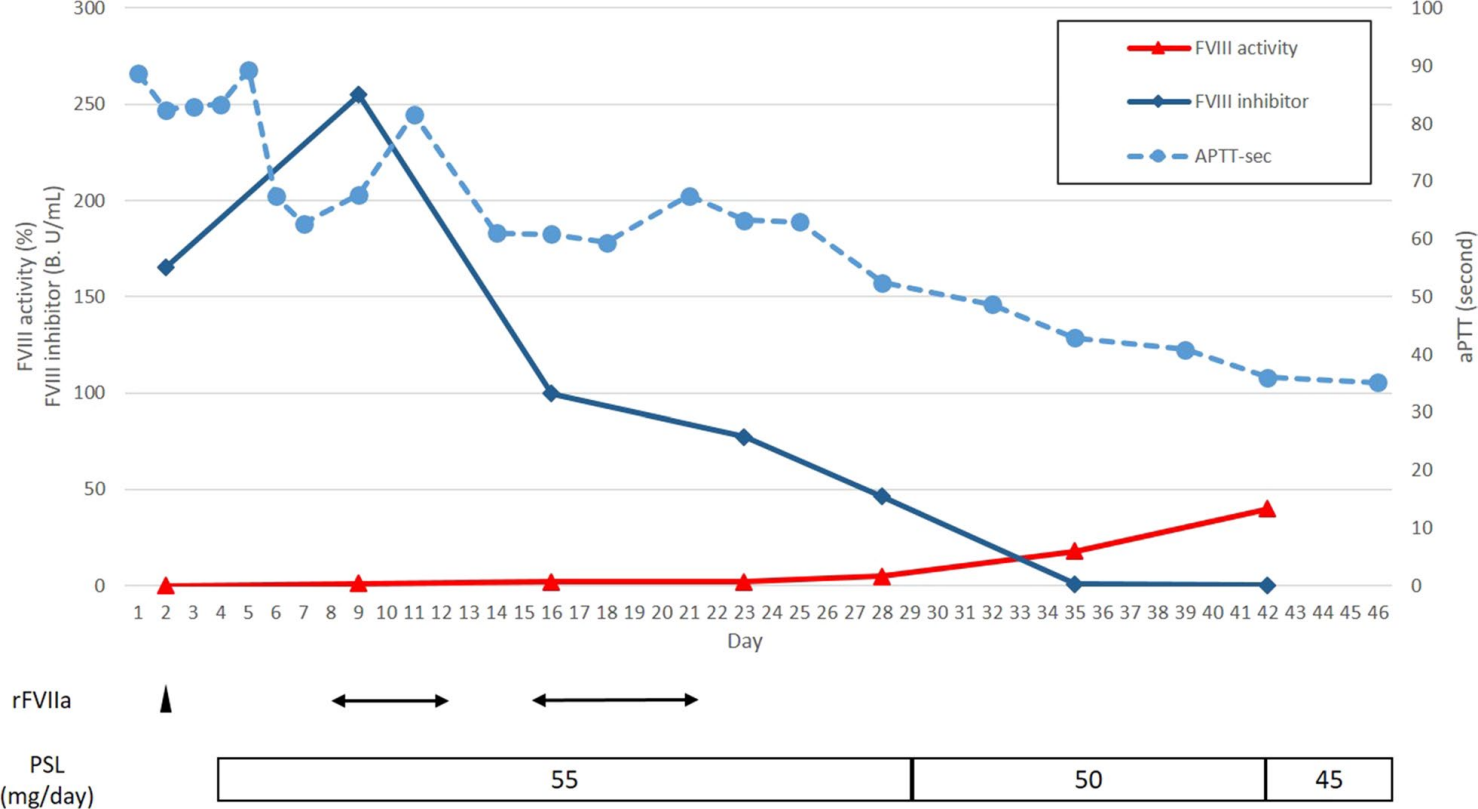
Hematological data during the hospital course. *aPTT* activated partial thromboplastin time, *rFVIIa* activated recombinant factor VII, *rFVIII* recombinant factor VIII

Postoperatively, the patient was admitted to the surgical intensive care unit with intubation, because of the possibility of epistaxis on removal of the endotracheal tube. Further, postoperatively, a few new hematomas < 10 mm in diameter arose on both sides of the velum at a different site from the surgical site (Fig. 4). These hematomas were far from the patient’s teeth and tongue; therefore, they were left in place. Although epistaxis was observed, after consulting with the hematologist and otolaryngologist, the nasotracheal tube was removed after administration of recombinant activated factor VII (rFVIIa) because AHA was suspected. Thereafter, fiberoptic examination was performed through the airway to confirm the absence of hemorrhage in the upper airway. Cross-mixing test showed a pattern of deficiency rather than a typical pattern of a coagulation factor inhibitor; therefore, initiation of immunosuppressive treatment was withheld. A few days after the surgery, purpura in the right upper and lower extremities that was not observed preoperatively became visible (Fig. 5). Blood transfusion and administration of rFVIIa were performed when purpura was observed or when the hemoglobin levels were < 6.0 g/dl. Abdominal CT and laboratory tests showed no evidence of malignancy or autoimmune disease. The dose of the factor VIII inhibitor was 165 Bethesda units at 7 days after surgery, following which a definitive diagnosis of AHA was made. The immunosuppressive treatment for eradication of the inhibitor was initiated with 55 mg/day of orally administered prednisolone. Gradually, anemia improved and the inhibitor level decreased (Fig. 6). The patient was discharged from the hospital on day 46. Oral administration of prednisolone 45 mg/day and follow-up examination were suggested.

## Discussion

Some case reports of AHA have shown the presence of interstitial hematomas, such as sublingual hematomas, which were treated conventionally for hemostasis; however, there have been no reports on exophytic hematomas in the oral cavity. We present the case of a patient with exophytic hematomas associated with AHA that were subjected to surgical hemostasis. To date, no reliable treatment for AHA has been recommended; therefore, patients are treated empirically according to the treatment established for congenital hemophilia [10]. In surgeries where patients with AHA are subjected to general anesthesia, orotracheal intubation is often difficult to perform because of low visibility in cases where hematomas arise in the oral cavity [11, 12]. Nasotracheal intubation with fiberoptic bronchoscopy is performed in such cases, except for cases with an active hemorrhage that may lead to a blind fiberoptic view [12]. Here, we examined how fiberoptic bronchoscopy could help avoid traumatic intubations. Notably, extubation was successfully completed with no complications. Interestingly, invasive procedures should be postponed until the eradication of the inhibitor, except for emergency situations [13]. It is necessary to consider that hemorrhagic diathesis in congenital hemophilia is associated with the factor VIII levels of anticoagulant factor; however in cases of AHA, there is no correlation with the level of anticoagulant factor [4, 5]. Although a coagulopathy was suspected, we performed emergency surgical hemostasis successfully, as the patient was at risk for severe hemorrhage from the large hematomas occupying the oral cavity.

To date, there are two major treatments, namely hemostasis and immunosuppression, to eradicate a coagulation inhibitor from a patient’s body [2, 13]. For treatment with a hemostatic agent, bypass treatments are used with rFVIIa or activated prothrombin complex concentrate. Evidence of a safe hemostasis technique is insufficient when invasive procedures are required [10]. Prednisolone or immunosuppressive agents such as cyclophosphamide are typically used to eradicate the inhibitor [14]. Eradication of the inhibitor takes approximately 5 weeks using only prednisolone [4]. Undertaking measures for infection prophylaxis is extremely important, because many deaths are caused by severe hemorrhage and infections associated with immunosuppressive agents [3, 7]. Moreover, patients with AHA are generally old. In this report, the patient’s past medical history included diabetes mellitus; therefore, to avoid the risk of infection, he was treated for AHA with oral prednisone alone and did not receive any other immunosuppressive agents.

Although the results of the aPTT cross-mixing test indicated a deficiency in the anticoagulant factor level rather than an inhibitor pattern typically presented in cases of AHA, AHA was strongly suspected because of the clinical manifestations. Therefore, prednisolone was administered before the detection of the inhibitor. AHA should be suspected in cases of prolonged aPTT and its clinical features. The differential diagnoses indicated the presence of von Willebrand disease and lupus anticoagulant, and suggested the use of heparin [14].

## Conclusion

The oral and maxillofacial regions include complex anatomical and highly vascular structures. Oral and maxillofacial surgeries, including soft and hard tissues, are performed in a wide range of operations. Hemorrhage in the oral cavity is one of the most severe complications because it causes airway obstruction. A maxillofacial surgeon must be familiar with hemorrhagic disorders, proper hemostasis techniques, and management procedures [5].

## Data Availability

Data sharing is not applicable to this article as no data sets were generated or analyzed during the current study.

## Abbreviations

AHA: Acquired hemophilia A
CT: Computed tomography
FVIII: Factor VIII
aPTT: Activated partial thromboplastin time
rFVIIa: Recombinant activated factor VII
